# Crystal structure of 1,3-bis­(2,3-di­methyl­quinoxalin-6-yl)benzene

**DOI:** 10.1107/S2056989015020435

**Published:** 2015-11-04

**Authors:** Charles E. Diesendruck, Gabrielle Rubin, Jeffery A. Bertke, Danielle L. Gray, Jeffrey S. Moore

**Affiliations:** a Department of Chemistry, University of Illinois, Urbana, Illinois 61801, USA; b University of Illinois, School of Chemical Sciences, Box 59-1, 505 South Mathews Avenue, Urbana, Illinois 61801, USA

**Keywords:** crystal structure, quionoxaline, Suzuki coupling, iridium catalyzed

## Abstract

The title compound, C_26_H_22_N_4_ (I), was synthesized by C—H iridium-catalyzed borylation followed by Suzuki coupling. The mol­ecular structure of (I) consists of a central benzene ring with 3-di­methyl­quinoxalin-6-yl groups at the 1 and 3 positions. These 2,3-di­methyl­quinoxalin-6-yl groups twist significantly out of the plane of the benzene ring. There are inter­molecular π–π inter­actions which result in a two-dimensional extended structure. The layers extend parallel to the *ab* plane and stack along the *c* axis.

## Abstract 2

The mol­ecular structure of the title complex is composed of a central benzene ring with 3-di­methyl­quinoxalin-6-yl groups at the 1 and 3 positions. There are inter­molecular π–π inter­actions which result in a two-dimensional extended structure. The layers extend parallel to the *ab* plane and stack along the *c* axis.

## Chemical context   

The title complex, (I), is one of the 1^st^ generation of quionoxaline-terminated polyphenyl­ene dendrimers that were prepared to study the effect of multivalency on the electrochemistry of quinoxalines (Carino *et al.*, 2015 ▸). The synthesis is based on C—H iridium-catalyzed borylation (Cho *et al.*, 2002 ▸) followed by Suzuki coupling, which was previously used in our group in the preparation of polyphenyl­ene dendrimers (Finke & Moore, 2008 ▸).
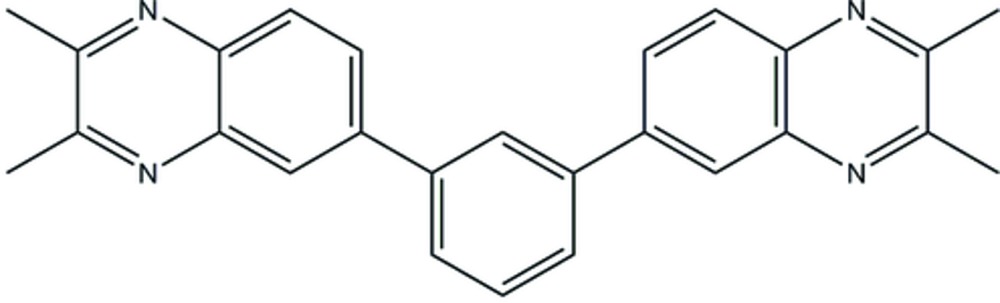


## Structural commentary   

**Figure 1:**
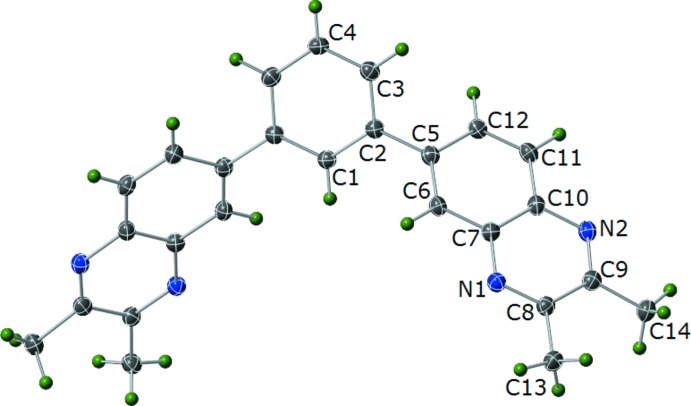
Plot showing 35% probability displacement ellipsoids for non-H atoms and circles of arbitrary size for H atoms for (I). The unlabeled atoms are related by the symmetry operator (−*x* + 1, *y*, −*z* + 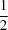).

The mol­ecular structure of (I) (Fig. 1 ▸) consists of a central phenyl ring with 2,3-di­methyl­quinoxalin-6-yl groups at the 1 and 3 positions. The C1 and C4 carbon atoms of the central phenyl ring each occupy special positions (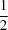, *y*, 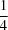) and thus one-half of the mol­ecule is generated by the symmetry operation (−*x* + 1, *y*, −*z* + 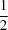). The 2,3-di­methyl­quinoxalin-6-yl group is twisted significantly out of the plane of the central phenyl ring as evidenced by the C1—C2—C5—C6 torsion angle of −39.8 (2)°. The two six-membered rings of the 2,3-di­methyl­quinoxalin-6-yl group deviate from planarity as well; the dihedral angle between a best fit plane through the C5–C6–C7–C10–C11–C12 ring and a best fit plane through the C7–N1–C8–C9–N2–C10 ring is 3.8 (15)°. The methyl groups also lie slightly out of the plane of the C7–N1–C8–C9–N2–C10 ring [N1–C8–C9–C14, τ = −176.41 (16)°; N2–C9–C8–C13, τ = −176.95 (15)°]. Similarly, the two methyl groups are not quite coplanar with a C13—C8—C9—C14 torsion angle of 3.5 (2)°.

## Supra­molecular features   

**Figure 2:**
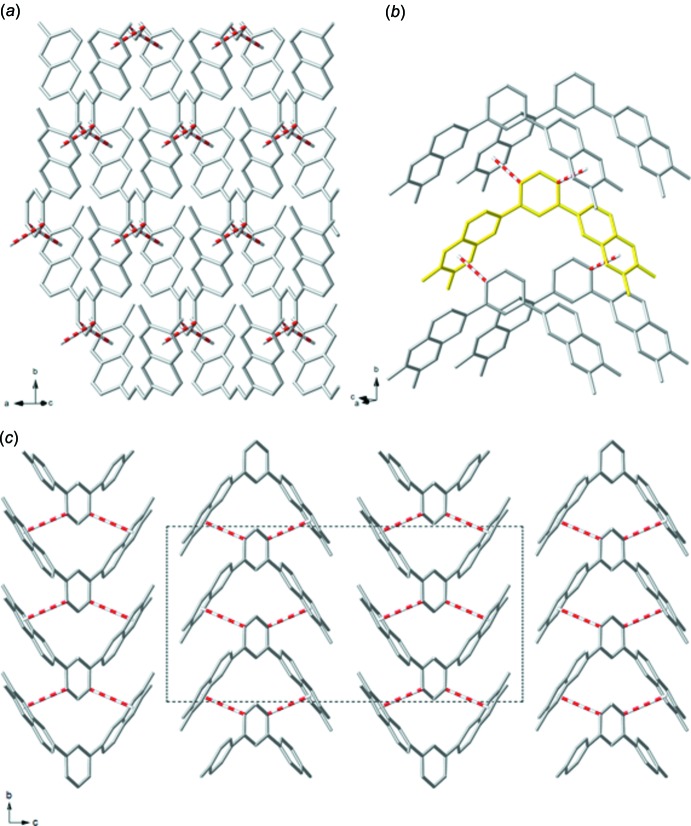
A plot of (*a*) a two-dimensional layer of (I), (*b*) a mol­ecule of (I) highlighted in yellow showing it inter­acting with its four nearest neighbors, and (*c*) a view along the *a* axis showing the separation between the layers and an overlay of the unit cell. All H atoms have been omitted for clarity. The inter­molecular inter­actions are indicated by red dashed lines.

The mol­ecules of (I) form extended layers *via* inter­molecular π–π inter­actions linking each mol­ecule to its four nearest neighbors, Fig. 2 ▸ *a*,*b*. The two-dimensional layers lie parallel to the *ab*-plane and stack along the *c* axis, Fig. 2 ▸ *c*. The inter­actions occur between the central benzene ring and one of the heterocycles on a neighboring mol­ecule. The orientation of these inter­acting groups is between ‘parallel offset’ and ‘perpendicular t-shaped’ as the C3—H3*A* bond points towards the C7^ii^–N1^ii^–C8^ii^–C9^ii^–N2^ii^–C10^ii^ ring centroid [symmetry code: (ii) *x* + 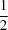, *y* + 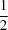, *z*]. The dihedral angle between a best fit plane through the C1–C2–C3–C4–C3^i^–C2^i^ [symmetry code: (i) −*x* + 1, *y*, −*z* + 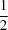] ring and a best-fit plane through the C7^ii^–N1^ii^–C8^ii^–C9^ii^–N2^ii^–C10^ii^ ring is 41.70 (11)°. The distance between the centroid of C7^ii^–N1^ii^–C8^ii^–C9^ii^–N2^ii^–C10^ii^ ring and C3 is 3.311 (3)Å. The centroid(C7–N1–C8–C9–N2–C10)⋯centroid(C7–N1–C8–C9–N2–C10) distance between the layers of 4.721 (3)Å is too long to be considered another π–π inter­action. It appears the methyl groups on the quinoxaline prevent the layers from coming closer together.

## Database survey   

A search of the Cambridge Crystal Database (Groom & Allen, 2014 ▸) returns zero results for 2,3-di­methyl­quinoxalin-6-yl groups attached to a phenyl ring. There are five reported crystal structures of 2,3-di­methyl­quinoxaline; the unsolvated species (Wozniak *et al.*, 1993 ▸), the di­methyl­glyoxime co-crystal (Hökelek *et al.*, 2001 ▸; Radhakrishnan *et al.*, 2007 ▸), the 2,6-di­hydroxy­toluene co-crystal, and the 2,6-di­hydroxy­toluene/4-di­methyl­amino­pyridine co-crystal (Mir *et al.*, 2015 ▸). A related compound, 2,3-dimethyl-6-nitro­quinoxaline, has been reported (Ghalib *et al.*, 2010 ▸) in which there is a nitro group bonded to the six-membered carbon ring of the quinoxaline. The dimeric version has also been characterized crystallographically, 2,2′,3,3′-tetra­methyl-6,6′-biquinoxaline, in which a single bond between the two six-membered carbon rings links a pair of 2,3-di­methyl­quinoxaline mol­ecules (Salvatore *et al.*, 2006 ▸).

The five 2,3-di­methyl­quinoxaline structures have a range of the dihedral angle between a best-fit plane through the six-membered carbon ring and a best-fit plane through the six-membered nitro­gen heterocycle of 0.02 (5)–1.59 (7)°. The two crystallographically independent mol­ecules of the nitro-substituted compound have dihedral angles of 0.18 (3) and 1.07 (2)°, while this angle is 4.93 (2)° for the dimeric complex. The methyl groups for all of these mol­ecules lie slightly out of the plane of the heterocycle with a range of N—C—C—Me torsion angles of 176.64 (7)–179.90 (5)°. The methyl groups in the database compounds range from nearly coplanar [Me—C—C—Me, τ = 0.09 (11)°] to significantly more twisted out of plane [Me—C—C—Me, τ = 3.33 (5)°]. Similar to (I), the dimeric mol­ecule deviates significantly from being a planar mol­ecule with a C2—C1—C1^iii^—C2^iii^ [symmetry code: (iii) −*x*, *y*, 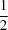 − *z*] torsion angle of −43.40 (10)° between the two 2,3-di­methyl­quinoxaline moieties.

## Synthesis and crystallization   

**Figure 3:**
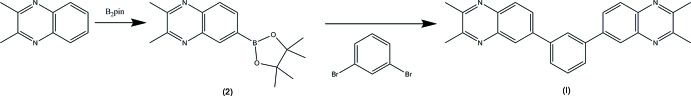
Reaction scheme.

Compound (I) was synthesized through the inter­mediate 2,3-dimethyl-6-(4,4,5,5-tetra­methyl-1,3,2-dioxaborolan-2-yl)quinoxaline (**2**) (see Fig. 3 ▸). In an Ar-filled dry box, a flame-dried vial with stirbar was charged with 2,3-dimethyl quinoxaline (349.1 mg, 2.21 mmol), bis(pinacolato)diboron B_2_pin] (423.0 mg, 1.67 mmol), [Ir(COD)(OMe)]_2_ (44 mg, 0.07 mmol), dtbpy (37 mg, 0.14 mmol) and cyclo­hexane (10 ml). The mixture was stirred inside the glovebox at 363 K for 4.5 h. Then, B_2_pin (427.0 mg, 1.68 mmol), [Ir(COD)(OMe)]_2_ (47 mg, 0.07 mmol), dtbpy (39 mg, 0.14 mmol) was added and the mixture further mixed at 363 K for 15 h. The reaction was filtered through silica, and the silica washed with chloro­form. The combined filtrate was evaporated and the product was purified by silica chromatography using 5% EA in hexane. (417.4 mg, 1.47 mmol) 88% yield. ^1^H NMR (400 MHz, CDCl­_3_): δ (p.p.m.) 8.42 (*s*, 1H), 7.96 (*d*, *J* = 9.6 Hz, 1H), 7.88 (*d*, *J* = 9.6 Hz, 1H), 2.65 (*s*, 3H), 2.64 (*s*, 3H), 1.31 (*s*, 12H).

A vial with a stirbar was charged with (**2**) (200 mg, 0.70 mmol), 1,3-di­bromo­benzene (70 mg, 0.30 mmol), Pd(OAc)_2_ (3.3 mg, 0.015 mmol), S-phos (12 mg, 0.03 mmol), THF (2 mL) and 5*M* NaOH (0.5 mL). The vial was sealed and heated to 343 K for 3 h. The solution was cooled and partitioned between Et_2_O (10 mL) and water (10 mL). The aqueous layer was extracted with Et_2_O (2 × 10 mL), the combined organic layers were washed with water and brine, and dried over anhydrous MgSO_4_. Column chromatography on silica gel eluting with 8:2 hexa­ne:EA to provide (I) (312.2 mg). Suitable single crystals were grown from slow diffusion of hexa­nes into a di­chloro­methane solution of (I). ^1^H NMR (400 MHz, CDCl­_3_): δ (p.p.m.) 8.29 (*s*, 2H), 8.09 (*d*, *J* = 9.6 Hz, 2H), 8.04 (*d*, *J* = 9.6 Hz, 2H), 8.03 (*s*, 1H), 7.80 (*d*, *J* = 7 Hz, 2H), 7.63 (*t*, *J* = 7 Hz, 1H), 2.77 (*s*, 12H). ^13^C NMR (100 MHz, CDCl­_3_): δ (p.p.m.) 154.1, 153.6, 141.4, 141.2, 140.8, 140.6 129.8, 128.8, 128.4, 127.0, 126.7, 126.2, 23.4, 23.3. MS–ESI (*m*/*z*): calculated for C_26_H_23_N_4_ [*M* + H]^+^: 391.2 found: 391.2.

## Refinement   

Crystal data, data collection and structure refinement details are summarized in Table 1 ▸. A structural model consisting of half of the target mol­ecule per asymmetric unit was developed. Methyl H atom positions, *R–*-CH_3_, were optimized by rotation about *R*—C bonds with idealized C—H, *R*—H and H—H distances. The remaining H atoms were included as riding idealized contributors. For methyl H atoms *U*_iso_(H) = 1.5*U*_eq_(C); *U*_iso_(H) = 1.2*U*_eq_(C) for remaining H atoms. The reflection 0 0 2 was omitted from the final refinements because it was partially blocked by the beamstop.

## Supplementary Material

Crystal structure: contains datablock(s) I. DOI: 10.1107/S2056989015020435/bg2571sup1.cif

Structure factors: contains datablock(s) I. DOI: 10.1107/S2056989015020435/bg2571Isup2.hkl

Supporting information file. DOI: 10.1107/S2056989015020435/bg2571Isup3.cdx

Supporting information file. DOI: 10.1107/S2056989015020435/bg2571Isup4.pdf

Supporting information file. DOI: 10.1107/S2056989015020435/bg2571Isup5.cml

CCDC reference: 1433738

Additional supporting information:  crystallographic information; 3D view; checkCIF report

## Figures and Tables

**Table 1:** Experimental details

Crystal data	
Chemical formula	C_26_H_22_N_4_
*M*_r_	390.47
Crystal system, space group	Monoclinic, *C*2/*c*
Temperature (K)	173
*a*, *b*, *c* (Å)	6.828 (3), 11.837 (5), 24.079 (11)
β (°)	91.902 (5)
*V* (Å^3^)	1945.0 (15)
*Z*	4
Radiation type	Mo *K*α
μ (mm^−1^)	0.08
Crystal size (mm)	0.30 × 0.17 × 0.17

Data collection	
Diffractometer	Siemens Platform/APEXII CCD
Absorption correction	Integration (*SADABS*; Bruker, 2014 ▸)
*T*_min_, *T*_max_	0.645, 1.000
No. of measured, independent and observed [*I* > 2σ(*I*)] reflections	7176, 1955, 1356
*R*_int_	0.066
(sin θ/λ)_max_ (Å^−1^)	0.623

Refinement	
*R*[*F* ^2^ > 2σ(*F* ^2^)], *wR*(*F* ^2^), *S*	0.047, 0.138, 1.04
No. of reflections	1955
No. of parameters	140
H-atom treatment	H-atom parameters not refined
Δρ_max_, Δρ_min_ (e Å^−3^)	0.23, −0.22

Computer programs: *APEX2*, *SAINT* (Bruker, 2014 ▸) and *XPREP* and *XCIF* (Bruker, 2014 ▸), *SHELXTL* (Sheldrick, 2008 ▸), *CrystalMaker* (CrystalMaker, 1994 ▸) and *publCIF* (Westrip, 2010 ▸).

## supplementary crystallographic information

### Crystal data

**Table:**

C_26_H_22_N_4_	*F*(000) = 824
*M**_r_* = 390.47	*D*_x_ = 1.333 Mg m^−^^3^
Monoclinic, *C*2/*c*	Mo *K*α radiation, λ = 0.71073 Å
Hall symbol: -C 2yc	Cell parameters from 1141 reflections
*a* = 6.828 (3) Å	θ = 3.4–26.1°
*b* = 11.837 (5) Å	µ = 0.08 mm^−^^1^
*c* = 24.079 (11) Å	*T* = 173 K
β = 91.902 (5)°	Prism, orange
*V* = 1945.0 (15) Å^3^	0.30 × 0.17 × 0.17 mm
*Z* = 4	

### Data collection

**Table:**

Siemens Platform/APEXII CCD diffractometer	1955 independent reflections
Radiation source: normal-focus sealed tube	1356 reflections with *I* > 2σ(*I*)
Graphite monochromator	*R*_int_ = 0.066
profile data from φ and ω scans	θ_max_ = 26.3°, θ_min_ = 3.4°
Absorption correction: integration (*SADABS*; Bruker, 2014)	*h* = −8→8
*T*_min_ = 0.645, *T*_max_ = 1.000	*k* = −14→14
7176 measured reflections	*l* = −29→29

### Refinement

**Table:**

Refinement on *F*^2^	Hydrogen site location: inferred from neighbouring sites
Least-squares matrix: full	H-atom parameters not refined
*R*[*F*^2^ > 2σ(*F*^2^)] = 0.047	*w* = 1/[σ^2^(*F*_o_^2^) + (0.060*P*)^2^ + 0.4908*P*] where *P* = (*F*_o_^2^ + 2*F*_c_^2^)/3
*wR*(*F*^2^) = 0.138	(Δ/σ)_max_ < 0.001
*S* = 1.04	Δρ_max_ = 0.23 e Å^−^^3^
1955 reflections	Δρ_min_ = −0.22 e Å^−^^3^
140 parameters	Extinction correction: *SHELXTL* (Bruker, 2014), Fc^*^=kFc[1+0.001xFc^2^λ^3^/sin(2θ)]^-1/4^
0 restraints	Extinction coefficient: 0.0082 (13)

### Special details

**Table:**

Experimental. One distinct cell was identified using *APEX2* (Bruker, 2014). Six frame series were integrated and filtered for statistical outliers using *SAINT* (Bruker, 2014) and then the combined data was corrected for absorption by integration, sorted, merged and scaled using *SADABS* (Bruker, 2014). No decay correction was applied.
Geometry. All e.s.d.'s (except the e.s.d. in the dihedral angle between two l.s. planes) are estimated using the full covariance matrix. The cell e.s.d.'s are taken into account individually in the estimation of e.s.d.'s in distances, angles and torsion angles; correlations between e.s.d.'s in cell parameters are only used when they are defined by crystal symmetry. An approximate (isotropic) treatment of cell e.s.d.'s is used for estimating e.s.d.'s involving l.s. planes.
Refinement. Structure was phased by direct methods. Systematic conditions suggested the ambiguous space group. The space group choice was confirmed by successful convergence of the full-matrix least-squares refinement on *F*^2^. The final difference Fourier map had no significant features. A final analysis of variance between observed and calculated structure factors showed little dependence on amplitude or resolution.

### Fractional atomic coordinates and isotropic or equivalent isotropic displacement parameters (Å^2^)

**Table:**

	*x*	*y*	*z*	*U*_iso_*/*U*_eq_	
N1	0.8418 (2)	0.49563 (11)	0.40609 (6)	0.0291 (4)	
N2	1.2363 (2)	0.57112 (12)	0.40420 (6)	0.0287 (4)	
C1	0.5000	0.75167 (19)	0.2500	0.0262 (6)	
H1A	0.5000	0.6714	0.2500	0.031*	
C2	0.6402 (2)	0.80925 (14)	0.28278 (7)	0.0265 (4)	
C3	0.6397 (3)	0.92738 (14)	0.28160 (7)	0.0294 (4)	
H3A	0.7363	0.9680	0.3027	0.035*	
C4	0.5000	0.9858 (2)	0.2500	0.0297 (6)	
H4A	0.5000	1.0661	0.2500	0.036*	
C5	0.7892 (2)	0.74799 (14)	0.31783 (7)	0.0273 (4)	
C6	0.7440 (3)	0.65150 (13)	0.34720 (7)	0.0278 (4)	
H6A	0.6127	0.6250	0.3469	0.033*	
C7	0.8910 (3)	0.59216 (13)	0.37760 (7)	0.0266 (4)	
C8	0.9866 (3)	0.43970 (14)	0.43105 (7)	0.0285 (4)	
C9	1.1886 (3)	0.47661 (14)	0.42908 (7)	0.0286 (4)	
C10	1.0870 (2)	0.63148 (14)	0.37843 (7)	0.0272 (4)	
C11	1.1300 (3)	0.73199 (14)	0.35013 (7)	0.0290 (4)	
H11A	1.2601	0.7605	0.3511	0.035*	
C12	0.9854 (3)	0.78862 (14)	0.32128 (7)	0.0289 (4)	
H12A	1.0165	0.8571	0.3030	0.035*	
C13	0.9366 (3)	0.33468 (15)	0.46213 (8)	0.0343 (5)	
H13A	0.7939	0.3282	0.4641	0.051*	
H13B	0.9950	0.3384	0.4998	0.051*	
H13C	0.9881	0.2687	0.4428	0.051*	
C14	1.3495 (3)	0.40683 (15)	0.45522 (8)	0.0355 (5)	
H14A	1.4766	0.4374	0.4449	0.053*	
H14B	1.3373	0.3286	0.4422	0.053*	
H14C	1.3399	0.4088	0.4957	0.053*	

### Atomic displacement parameters (Å^2^)

**Table:**

	*U*^11^	*U*^22^	*U*^33^	*U*^12^	*U*^13^	*U*^23^
N1	0.0268 (8)	0.0301 (8)	0.0305 (8)	−0.0014 (6)	0.0008 (7)	0.0005 (6)
N2	0.0240 (8)	0.0340 (8)	0.0280 (8)	0.0010 (6)	0.0013 (7)	−0.0021 (6)
C1	0.0236 (13)	0.0262 (12)	0.0290 (13)	0.000	0.0039 (11)	0.000
C2	0.0260 (10)	0.0298 (9)	0.0239 (9)	−0.0005 (7)	0.0050 (8)	0.0003 (7)
C3	0.0315 (11)	0.0305 (9)	0.0262 (9)	−0.0027 (7)	0.0015 (8)	−0.0030 (7)
C4	0.0360 (15)	0.0255 (12)	0.0278 (13)	0.000	0.0034 (12)	0.000
C5	0.0278 (10)	0.0297 (9)	0.0243 (9)	0.0006 (7)	−0.0004 (8)	−0.0031 (7)
C6	0.0219 (9)	0.0319 (9)	0.0295 (10)	−0.0019 (7)	0.0001 (8)	−0.0010 (7)
C7	0.0269 (10)	0.0292 (9)	0.0238 (9)	0.0000 (7)	0.0034 (8)	−0.0007 (7)
C8	0.0299 (10)	0.0289 (9)	0.0263 (9)	0.0012 (8)	−0.0016 (8)	−0.0017 (7)
C9	0.0304 (10)	0.0321 (9)	0.0233 (9)	0.0029 (7)	0.0001 (8)	−0.0047 (7)
C10	0.0241 (10)	0.0316 (9)	0.0257 (9)	0.0000 (7)	−0.0003 (8)	−0.0037 (7)
C11	0.0243 (10)	0.0339 (10)	0.0289 (10)	−0.0050 (7)	0.0024 (8)	−0.0037 (7)
C12	0.0291 (10)	0.0317 (9)	0.0258 (9)	−0.0032 (7)	0.0022 (8)	−0.0001 (7)
C13	0.0338 (11)	0.0333 (10)	0.0355 (11)	0.0010 (8)	−0.0029 (9)	0.0049 (8)
C14	0.0299 (11)	0.0392 (10)	0.0371 (11)	0.0045 (8)	−0.0015 (9)	0.0015 (8)

### Geometric parameters (Å, º)

**Table:**

N1—C8	1.318 (2)	C6—H6A	0.9500
N1—C7	1.380 (2)	C7—C10	1.416 (2)
N2—C9	1.315 (2)	C8—C9	1.449 (3)
N2—C10	1.376 (2)	C8—C13	1.496 (2)
C1—C2	1.3980 (19)	C9—C14	1.496 (2)
C1—C2^i^	1.3980 (19)	C10—C11	1.407 (2)
C1—H1A	0.9500	C11—C12	1.364 (2)
C2—C3	1.399 (2)	C11—H11A	0.9500
C2—C5	1.488 (2)	C12—H12A	0.9500
C3—C4	1.385 (2)	C13—H13A	0.9800
C3—H3A	0.9500	C13—H13B	0.9800
C4—C3^i^	1.385 (2)	C13—H13C	0.9800
C4—H4A	0.9500	C14—H14A	0.9800
C5—C6	1.384 (2)	C14—H14B	0.9800
C5—C12	1.423 (2)	C14—H14C	0.9800
C6—C7	1.410 (2)		
			
C8—N1—C7	116.81 (15)	C9—C8—C13	120.00 (15)
C9—N2—C10	117.13 (15)	N2—C9—C8	121.45 (15)
C2—C1—C2^i^	121.6 (2)	N2—C9—C14	118.15 (16)
C2—C1—H1A	119.2	C8—C9—C14	120.40 (16)
C2^i^—C1—H1A	119.2	N2—C10—C11	119.60 (16)
C1—C2—C3	118.34 (16)	N2—C10—C7	121.43 (16)
C1—C2—C5	121.66 (16)	C11—C10—C7	118.91 (15)
C3—C2—C5	120.00 (15)	C12—C11—C10	120.21 (16)
C4—C3—C2	120.79 (16)	C12—C11—H11A	119.9
C4—C3—H3A	119.6	C10—C11—H11A	119.9
C2—C3—H3A	119.6	C11—C12—C5	121.83 (16)
C3^i^—C4—C3	120.1 (2)	C11—C12—H12A	119.1
C3^i^—C4—H4A	120.0	C5—C12—H12A	119.1
C3—C4—H4A	120.0	C8—C13—H13A	109.5
C6—C5—C12	118.32 (15)	C8—C13—H13B	109.5
C6—C5—C2	122.17 (16)	H13A—C13—H13B	109.5
C12—C5—C2	119.51 (16)	C8—C13—H13C	109.5
C5—C6—C7	120.66 (16)	H13A—C13—H13C	109.5
C5—C6—H6A	119.7	H13B—C13—H13C	109.5
C7—C6—H6A	119.7	C9—C14—H14A	109.5
N1—C7—C6	119.33 (16)	C9—C14—H14B	109.5
N1—C7—C10	120.71 (15)	H14A—C14—H14B	109.5
C6—C7—C10	119.96 (16)	C9—C14—H14C	109.5
N1—C8—C9	122.28 (16)	H14A—C14—H14C	109.5
N1—C8—C13	117.71 (16)	H14B—C14—H14C	109.5
			
C2^i^—C1—C2—C3	−0.93 (12)	C10—N2—C9—C8	−2.2 (2)
C2^i^—C1—C2—C5	179.81 (18)	C10—N2—C9—C14	177.32 (15)
C1—C2—C3—C4	1.9 (2)	N1—C8—C9—N2	3.1 (3)
C5—C2—C3—C4	−178.83 (14)	C13—C8—C9—N2	−176.95 (15)
C2—C3—C4—C3^i^	−0.97 (12)	N1—C8—C9—C14	−176.41 (16)
C1—C2—C5—C6	−39.8 (2)	C13—C8—C9—C14	3.5 (2)
C3—C2—C5—C6	140.92 (18)	C9—N2—C10—C11	−178.64 (15)
C1—C2—C5—C12	139.70 (15)	C9—N2—C10—C7	−1.4 (2)
C3—C2—C5—C12	−39.6 (3)	N1—C7—C10—N2	4.6 (3)
C12—C5—C6—C7	−3.0 (3)	C6—C7—C10—N2	−175.09 (15)
C2—C5—C6—C7	176.57 (15)	N1—C7—C10—C11	−178.22 (14)
C8—N1—C7—C6	176.03 (15)	C6—C7—C10—C11	2.1 (3)
C8—N1—C7—C10	−3.6 (2)	N2—C10—C11—C12	175.60 (15)
C5—C6—C7—N1	−179.42 (15)	C7—C10—C11—C12	−1.7 (3)
C5—C6—C7—C10	0.2 (3)	C10—C11—C12—C5	−1.1 (3)
C7—N1—C8—C9	0.0 (2)	C6—C5—C12—C11	3.5 (3)
C7—N1—C8—C13	−179.94 (14)	C2—C5—C12—C11	−176.07 (15)

Symmetry code: (i) −*x*+1, *y*, −*z*+1/2.
