# Supplementary figures and images for: Crystal structure of 1,3-bis­(2,3-di­methyl­quinoxalin-6-yl)benzene
Source: Acta Crystallogr E Crystallogr Commun. 2015 Nov 4;71(Pt 12):1429–32. doi: 10.1107/S2056989015020435 (PMC4719806; doi:10.1107/S2056989015020435)

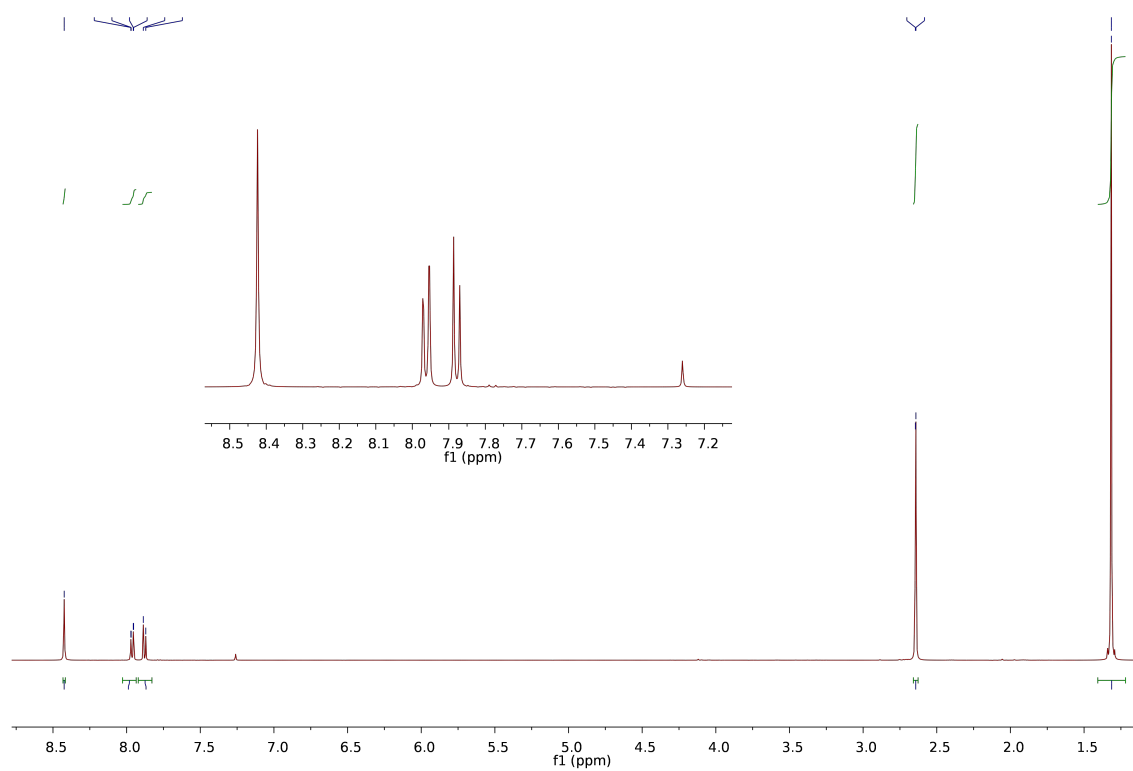

$^1\text{H}$  NMR spectrum of synthetic intermediate **(2)**.

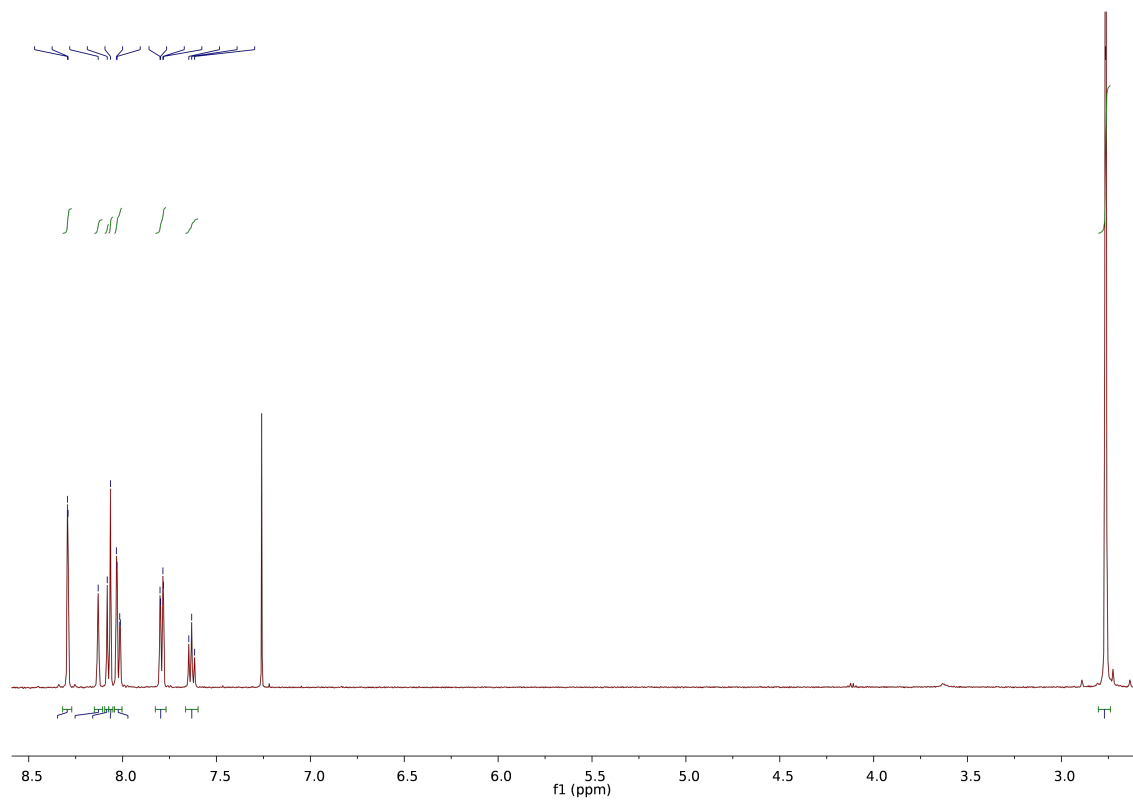

$^1\text{H}$  NMR spectrum of (I).

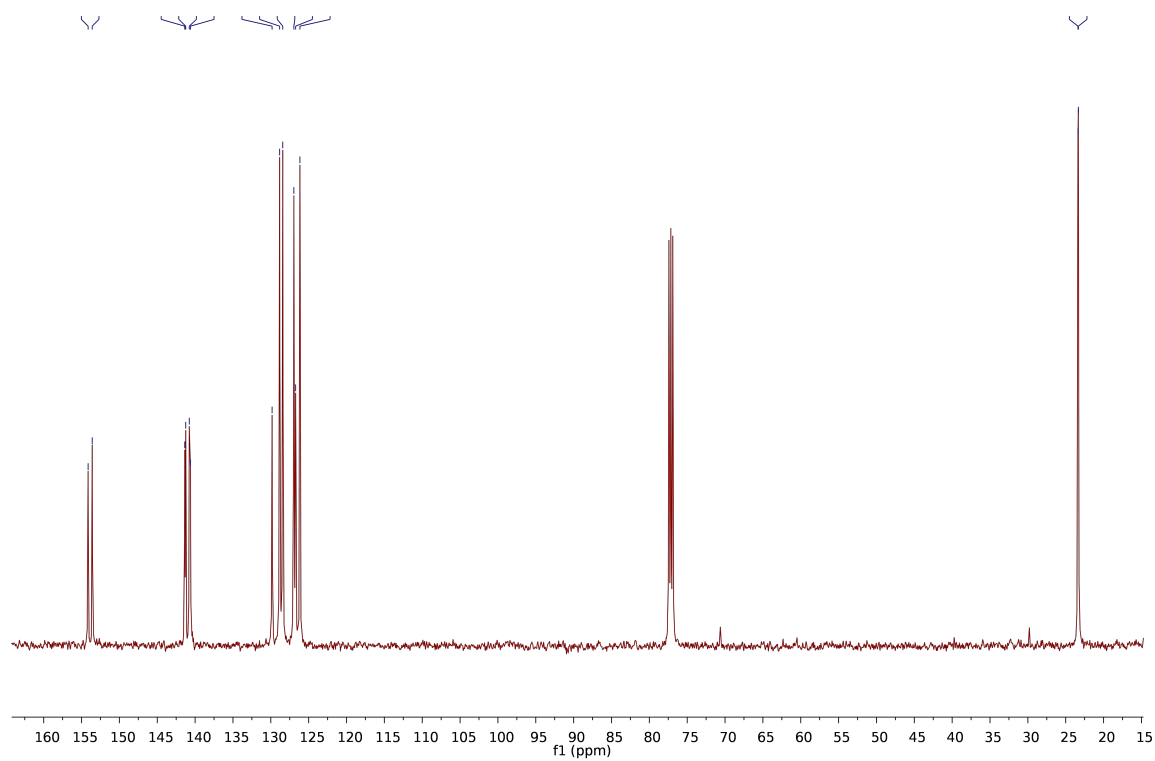

$^{13}\text{C}$  NMR spectrum of (I).

Supplement: Supplementary file 4 [file e-71-01429-Isup4.pdf]
